# Adherence to the 2019 AHA/ACC/HRS Focused Update of the 2014 AHA/ACC/HRS Guideline on the Use of Oral Anticoagulant Agents in Middle Eastern Patients with Atrial Fibrillation: The Jordan Atrial Fibrillation (JoFib) Study

**DOI:** 10.1155/2021/5515089

**Published:** 2021-04-08

**Authors:** Ayman J. Hammoudeh, Yousef Khader, Nazih Kadri, Eyas Al-Mousa, Yahya Badaineh, Laith Habahbeh, Ramzi Tabbalat, Hesham Janabi, Imad A. Alhaddad

**Affiliations:** ^1^Cardiology Department, Istishari Hospital, 44 Kindi Street Amman 11184, Jordan; ^2^Department of Public Health, Jordan University of Science and Technology School of Medicine, 3030 Ramtha Street, P.O. Box 3030, Irbid 22110, Jordan; ^3^Electrophysiology and General Cardiology Sections, Cardiology Department, Abdali Hospital, 1 Al-Istethmar Street, Abdali Boulevard, Amman 11190, Jordan; ^4^Intensive Care Unit, Istishari Hospital, 44 Kindi Street, Amman 11184, Jordan; ^5^Cardiology Department, Aqaba Isl Hospital, 15 Sharif Shaker Ben Zaidstreet, Aqaba 77110, Jordan; ^6^Cardiology Department, Jordan Hospital, 9 Nuzha Street, Amman 11196, Jordan

## Abstract

**Background:**

There is a scarcity of studies that evaluate adherence to the utilization of guideline-recommended oral anticoagulant agents (OACs) in patients with atrial fibrillation (AF) in the Middle East. The Jordan Atrial Fibrillation (JoFib) Study evaluated baseline clinical profiles and the utilization of OACs, including vitamin K antagonists (VKAs) and direct OACs (DOACs), in patients with valvular AF (VAF) and nonvalvular AF (NVAF) according to the 2019 focused update of the 2014 AHA/ACC/HRS guidelines.

**Methods:**

Consecutive patients with AF were enrolled in 29 hospitals and outpatient clinics. The use of OACs was evaluated in patients with VAF and NVAF according to the prespecified guideline.

**Results:**

Of 2000 patients, 177 (8.9%) had VAF and 1823 (91.1%) had NVAF. A VKA was prescribed for 88.1% of the VAF group. In the NVAF group, 1468 (80.5%) of patients had a high CHA_2_DS_2_-VASc score, i.e., a score of ≥3 in women and ≥2 in men; 202 (11.1%) patients had an intermediate CHA_2_DS_2_-VASc score, i.e., a score of 2 in women and 1 in men; and 153 (8.4%) patients had a low CHA_2_DS_2_-VASc score, i.e., a score of 1 in women and 0 in men. Of patients with a high CHA_2_DS_2_-VASc score, 1204 (82.0%) received OACs, including DOACs for 784 (53.4%) and VKA for 420 (28.6%) patients. Among patients with an intermediate score, OACs were prescribed for 148 (73.3%) patients, including 107 (53.0%) who received DOACs and 41 (20.3%) patients who received VKA. In patients with a low score, OACs were omitted in 94 (61.4%) patients and prescribed for 59 (38.6%) patients. Multivariate analysis showed that age between 50 and 70 years, CHA_2_DS_2_-VASc score of ≥2, a diagnosis of stroke or systemic embolization, and nonparoxysmal AF were significantly associated with increased odds of OAC prescription.

**Conclusions:**

The current status of the utilization of OACs in Middle Eastern AF patients appears to be promising and is consistent with the 2019 focused update of the 2014 AHA/ACC/HRS guideline. This trial is registered with NCT03917992.

## 1. Introduction

Atrial fibrillation (AF) is the most common sustained cardiac arrhythmia in adults [1, 2]. The most concerning health risks of AF are stroke and systemic embolization which are mostly preventable by the use of recommended oral anticoagulant agents (OACs) [3]. Vitamin K antagonists (VKAs) are the recommended OACs for patients with valvular AF (VAF) which accounts for 4% to 26% of all AF patients [4] and includes patients with moderate to severe rheumatic mitral valve stenosis and those with mechanical prosthetic valves [5]. On the other hand, direct OACs (DOACs) are recommended over VKA for patients with nonvalvular AF (NVAF) at high risk of stroke or systemic embolization [6, 7]. NVAF is associated with a wide range of etiologies including ischemic heart disease, hypertensive heart disease, nonrheumatic valve disease, and cardiomyopathies among other diseases [7]. There are significant heterogeneities in the etiology of AF, baseline clinical profiles of patients with VAF and NVAF, and utilization of OACs in different regions in the world due to discrepancies in age pyramids, prevalence of cardiovascular risk factors and comorbid diseases, and availability of and accessibility to emerging therapeutic agents [8–10]. Real-world AF registries have not only provided substantial evidence to supplement data from the randomized controlled trials comparing DOACs with VKA for prevention of stroke and systemic embolization but also serve as effective tools to examine patient characteristics, adherence to practice guidelines in the use of OACs, and long-term outcomes in patients with AF [8, 10–12].

Most clinical and epidemiological studies and registries of AF have been conducted in Western countries where clinical features, guideline adherence, and prognosis in patients with AF differ significantly compared with those in the Middle East [2, 13–15]. Studies from the Middle East have shown that the AF population is younger and has higher prevalence of cardiovascular risk factors and comorbid diseases including sedentary lifestyles, obesity, diabetes mellitus, hypertension, and coronary heart disease [16, 17]. Studies have also demonstrated a pattern of low rate of utilization of OACs in general and DOACs in particular compared with patients in the West [13, 17–19]. Major limitations of these studies restrict their applicability in wider Middle Eastern countries and populations. Two large regional AF studies [20, 21] enrolled an inhomogeneous cohort that included native as well as Southeast Asian patients. The Southeast Asian patients included in the Gulf registry were mostly foreign workers with different clinical profiles compared with the native population.

Furthermore, these studies did not evaluate the prevalence and demographic and clinical disparities between VAF and NVAF, and many of these studies were conducted before the widespread use of DOACs. Other limitations include the small sample size, retrospective design, single center experience, and lack of data on the adherence to recent clinical practice guidelines in the utilization of OACs according to the CHA_2_DS_2_-VASc score [16, 22–26].

The Jordan Atrial Fibrillation (JoFib) Study enrolled a consecutive cohort of patients with AF evaluated at a large number of hospitals and ambulatory care clinics in a Middle Eastern country to provide a contemporary insight on the baseline clinical features of patients with VAF and NVAF, presence of comorbid diseases, the CHA_2_DS_2_-VASc score, the utilization of OACs according to the 2019 American Heart Association (AHA)/American College of Cardiology (ACC)/Heart Rhythm Society (HRS) focused update of the 2014 AHA/ACC/HRS guideline for the management of patients with atrial fibrillation [6], and the independent factors associated with the use of these medications.

## 2. Materials and Methods

The JoFib Study is a prospective multicenter observational registry that enrolled consecutive patients aged ≥18 years who were diagnosed to have AF in 18 hospitals and 11 outpatient cardiology clinics from May 25, 2019, through October 25, 2020. Data were collected using a standardized clinical data form at the time of enrollment, and at one, 6, and 12 months after the initial assessment. Diagnosis of AF was confirmed by 12-lead electrocardiogram (EKG), rhythm strip lasting ≥30 seconds, ≥1 episodes of AF on ambulatory EKG monitor, or a past diagnosis by a treating cardiologist. Baseline data included clinical and demographic profiles, laboratory data, EKG, transthoracic echocardiographic features, and the use of OACs and other pharmacological medications. Standard definitions were used to classify the types of AF, including paroxysmal, persistent, long standing, and permanent [6], and to calculate the CHA_2_DS_2_-VASc [27] and HAS-BLED [28] scores for each patient. Eligibility for oral anticoagulant agents was analyzed based on the 2019 AHA/ACC/HRS focused update of the 2014 AHA/ACC/HRS guideline for the management of patients with atrial fibrillation [6]. This update recommends VKA for patients with VAF (i.e., those who have moderate to severe mitral stenosis or a mechanical heart valve) (Class I indication) and DOACs over warfarin in eligible patients with AF including women with a CHA_2_DS_2_-VASc score of ≥3 or men with a score of ≥2 (Class I recommendation). The update recommends considering the use of OACs in women with a CHA_2_DS_2_-VASc score of 2 and men with a score of 1 (Class IIb recommendation) and omitting OACs in women with a CHA_2_DS_2_-VASc score of 1 or men with a score of 0 (Class IIa recommendation). Patients with a contraindication to OACs at the time of study enrollment (active bleeding or high risk of bleeding) were considered ineligible for OAC use regardless of the CHA_2_DS_2_-VASc score.

The study was approved by the Institutional Review Board of participating centers, and patients signed written informed consent. All treatment decisions were left to the discretion of the treating physician. The study was registered with Clinicaltrials.gov (unique identifier number).

## 3. Statistical Analysis

Descriptive statistics were performed using means and standard deviation (SD) to describe the continuous variables, and percentages were used to describe the categorical variables. An independent *t*-test was used to compare means, and a chi-squared test was used to compare percentages. Multivariate binary logistic regression was conducted to determine factors associated with OAC use. The variables in the logistic regression model were selected using a stepwise backward method. *p* value of ≤0.05 was considered statistically significant.

## 4. Results and Discussion

### 4.1. Results

Of the 2000 consecutively enrolled patients, 177 (8.9%) patients had VAF and 1823 (91.1%) patients had NVAF. Patients in the VAF group had either moderate to severe mitral stenosis (66 patients, 37.3%) or mechanical prosthetic valve (111 patients, 62.7%). The baseline clinical characteristics of both groups are shown in Table 1. Compared with patients in the VAF group, patients in the NVAF group were older and had higher prevalence of the classical cardiovascular risk factors, except overweight which was more prevalent in patients with VAF. Prevalence of nine prespecified comorbid diseases was similar in the two groups, except ischemic heart disease which was more prevalent in the NVAF group compared to the VAF group. The echocardiographic data demonstrated a larger left atrial size and higher prevalence of pulmonary artery hypertension, but similar left ventricular ejection fraction in the VAF group compared to the NVAF group.

VKA was prescribed for the majority (156, 88.1%) of patients with VAF. DOACs were prescribed for 14 (7.9%) patients, and 7 (4.0%) patients were not prescribed with OACs.

**Figure 1:**
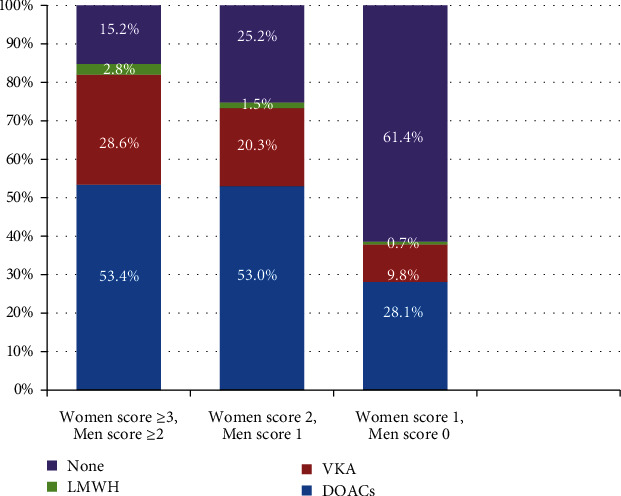
Use of anticoagulant agents in patients with nonvalvular AF according to the CHA_2_DS_2_-VASc score. DOACs: direct oral anticoagulant agents; LMWH: low molecular weight heparin; VKA: vitamin K antagonist.

The CHA_2_DS_2_-VASc score was used to determine the use of OACs in patients with NVAF (Figure 1). The mean CHA_2_DS_2_-VASc score in women was 4.2 ± 1.3 and in men 3.1 ± 1.7. There were 1468 (80.5%) patients with high scores (women with a CHA_2_DS_2_-VASc score of ≥3 and men with a score of ≥2). Of those, 1204 (82.0%) received OACs; DOACs were given to 784 (53.4%) and VKA to 420 (28.6%) patients.

Of 202 (11.1%) patients with an intermediate score, OACs were prescribed for 148 (73.3%) patients including 107 (53.0%) patients who received DOACs and 41 (20.3%) patients who received VKA. Finally, there were 153 (8.4%) patients with a low score, in whom OACs were omitted in 94 (61.4%) patients and prescribed for 59 (38.6%) patients. Among patients with NVAF, 368 (20.2%) did not receive OACs and 45 (2.4%) were prescribed with low molecular weight heparin. Contraindication for anticoagulation was documented in 41 of these patients including active bleeding, high risk of bleeding, or enrollment in the study during the perioperative period.

Aspirin, a second antiplatelet agent, and dual antiplatelet agents were prescribed for 755 (37.8%), 279 (14.0%), and 127 (6.4%) of all patients, respectively. There were 596 (29.8%) patients who were prescribed a combination of OAC and antiplatelet agent(s). Of 264 patients not receiving OACs despite high CHA_2_DS_2_-VASc scores, 190 (72.0%) were prescribed with antiplatelet agents.

In the univariate analysis (Table 2), OACs were more likely to be prescribed for females, patients aged 50 years or older, patients with hypertension or diabetes mellitus, nonsmokers, patients with nonparoxysmal AF, patients with prior stroke or systemic embolization, patients with left ventricular ejection fraction < 40%, patients with heart failure, and patients with a CHA_2_DS_2_-VASc score ≥ 2 or 3. In the multivariate analysis (Table 3), patients aged between 50 and 70 years were almost twice (OR = 1.8) more likely to be prescribed OACs compared to patients aged <50 years. The odds of OAC prescription were significantly higher among patients treated in outpatient clinics, nonsmokers, patients with dyslipidemia, patients with cancer, patients with stroke or systemic embolization, and patients with nonparoxysmal AF. A CHA_2_DS_2_-VASc score of ≥2 was significantlyassociated with increased odds of OAC prescription. A one-unit increase in the HAS-BLED score was significantly associated with a 20% decrease in the odds of OAC prescription.

## 5. Discussion

This study represents the first registry of a large cohort of Middle Eastern patients that focuses on contemporary management of AF according to the 2019 AHA/ACC/HRS focused update of the 2014 AHA/ACC/HRC guideline on the use of OACs for the prevention of the most serious and life-threatening complications, stroke, and systemic embolization [6]. The study showed that about 9 out of 10 of the enrolled patients have NVAF and also showed a high degree of adherence to current practice guidelines on OAC utilization, with more than 8 out of 10 of patients with NVAF who have a high CHA_2_DS_2_-VASc score received OACs with DOACs accounting for 65%.

Contrary to the belief of valvular heart disease, and rheumatic mitral stenosis in particular, being the main cause of AF in developing countries, this study concurs with other Middle Eastern studies showing that <10% of all patients with AF have VAF [21, 23, 29]. This could be a reflection of decreasing incidence and prevalence of rheumatic heart disease in our region and the increasing prevalence of cardiovascular risk factors and comorbidities that predispose to NVAF including the demographic transition to an inverted age pyramid with an increase in numbers of older population, hypertension, diabetes mellitus, coronary heart disease, obesity, and sedentary lifestyles [30–32]. The rising burden of NVAF, among other cardiovascular diseases in developing countries, reflects a rising prevalence of noncommunicable diseases and leads to an increase in total mortality, morbidity, and health budgets in the region [33, 34].

Despite these worrying trends, we lack studies that address the clinical profiles, utilizing OACs according to contemporary guidelines and factors affecting the prescription patterns in a large cohort of patients in this region. Smaller studies from the Middle East and other regions have demonstrated a significant geographical heterogeneity in rates of adherence to the published guidelines in prescribing DOACs vs. VKAs for patients with AF [35–37]. Utilization of OAC in our study was closer to those reported from Western countries where the rate of use of OACs ranged between 34% and 85% of patients [5, 8, 38, 39] with DOACs accounting for 41% of OACs [8]. The use of OACs in about one-third of patients with low CHA_2_DS_2_-VASc scores, despite a Class IIa recommendation to omit these agents, might be explained by an exaggerated perception by the treating physician of the risk of stroke or systemic embolization in such patients.

It is noteworthy that non-OAC antithrombotic medications were prescribed in this study for a small (15%) group of NVAF patients with high CHA_2_DS_2_-VASc scores; seven of 10 of those patients were prescribed one or two antiplatelet agent(s). Several studies [40, 41] have shown that antiplatelet agents are being prescribed to a considerable portion of OAC-eligible patients despite the lack of a protective role in patients with NVAF with high CHA_2_DS_2_-VASc [6]. The most recent clinical practice guideline on the management of AF by the European Society of Cardiology was published in mid-2020 [42], at a time when this study was in its final enrollment phases. This guideline is essentially not different from the 2019 update guideline we used for evaluating the adherence to OAC use.

The independent factors that were shown by this study to be associated with higher rates of OAC utilization are not different from those reported by other investigators [6, 8–10, 43, 44]. Patients who had or at higher risk of stroke or systemic embolization due to a high CHA_2_DS_2_-VASc score were more likely to be prescribed with DOACs than those who did not have these features. Patients aged between 50 and 75 years being more likely to receive OACs than those older than 75 years is probably related to perceived lower risk of bleeding in this age group compared with older patients. Another finding of the study is that a high bleeding risk, indicated by a higher HAS-BLED score, negatively impacted OAC utilization. This may account in part for gaps in adherence to AF management guidelines which explicitly state that a high level of HAS-BLED score is not a justification to withhold OAC [6, 42].

In line with other studies [44–46], we found that patients with nonparoxysmal AF were more likely to receive OACs than those with paroxysmal AF. It is well established that a significant proportion of episodes of stroke in patients with AF occur in those with a history of paroxysmal AF, and the guidelines recommend OAC for stroke prevention for all types of AF with appropriate scores.

Other factors reported by other investigators to be associated with higher odds of utilizing OACs include duration of AF > 5 years, higher education levels, treatment by a cardiologist, and using a pill sorting box and calendar. Frailty and self-paying, on the other hand, were associated with lower odds of prescribing OACs [43–45].

The present study has few potential limitations. Similar to all registries of observational nature, data collected might be subject to potential bias despite reinforcing consecutive recruitment from the outset of the study. The fact that this study was based on cardiologist-managed patients, selection bias may limit the generalizability of these findings to patients treated by other specialties. Involving 29 outpatient clinics and hospitals from public university and private sectors in the study will enhance the generalizability of the results. Despite these limitations, the data shown represent a contemporary addition to studies that evaluate clinical features and utilization of OACs in a Middle Eastern population with VAF and NVAF. Furthermore, the study sets a higher standard for adherence to recent clinical practice guidelines on the utilization of OACs. Participating centers will be provided with feedback to create a quality improvement initiative geared toward increasing the utilization of evidence-based pharmacotherapy for patients with AF and will help further dissemination of such measures to other specialties dealing with AF.

## 6. Conclusions

The study demonstrated an assuring level of adherence to the 2019 AHA/ACC/HRS focused update of the 2014 AHA/ACC/HRS guideline on the use of oral anticoagulant agents in Middle Eastern patients with VAF and NVAF. Impact of these findings on the incidence of acute stroke, systemic embolization, and bleeding events awaits the one-year follow-up data.

## Figures and Tables

**Table 1:** Baseline clinical characteristics of 2000 patients with valvular and nonvalvular atrial fibrillation.

Clinical features	Valvular AF (*N* = 177, 8.9%)	Nonvalvular AF (*N* = 1823, 91.1%)	*p* value
Age in years (mean ± SD)	62.1 ± 12.0	69.6 ± 11.4	<0.001
Age < 50 years	27 (15.3%)	165 (9.1%)	<0.001
Age > 75 years	18 (10.2%)	533 (29.2%)	<0.001
Females	118 (66.7%)	949 (52.1%)	<0.001
Hypertension	95 (53.7%)	1391 (76.3%)	<0.001
Diabetes mellitus	55 (31.1%)	827 (45.4%)	<0.001
Body mass index > 25 kg/m^2∗^	104 (65.8%)	1288 (77.3%)	0.002
Hypercholesterolemia	49 (27.7%)	829 (45.5%)	<0.001
Current cigarette smoking	26 (14.7%)	247 (13.6%)	0.65
Permanent atrial fibrillation	83 (46.9%)	521 (28.6%)	<0.001
Stroke or systemic embolization	27 (15.3%)	298 (16.3%)	0.83
Ischemic heart disease	13 (7.3%)	212 (11.6%)	<0.001
Heart failure	33 (18.6%)	434 (23.8%)	0.14
Left ventricular ejection fraction < 40%^∗^	16 (9.2%)	229 (13.4%)	0.15
Pulmonary hypertension^∗^	79 (44.6%)	264 (25.5%)	<0.001
Left ventricular hypertrophy^∗∗^	54 (33.8%)	653 (39.6%)	0.09
Sleep apnea	3 (17%)	73 (4.0%)	0.15
Chronic lung disease	6 (3.4%)	79 (4.3%)	0.64
Chronic kidney disease	11 (6.2%)	170 (9.3%)	0.22
HAS-BLED score (mean ± SD)	1.5 ± 1.2	1.7 ± 1.1	0.004

^∗^Body mass index was measured in 1824 patients, left ventricular ejection fraction in 1888 patients, pulmonary hypertension in 1998 patients, and left ventricular hypertrophy in 1808 patients.

**Table 2:** Univariate analysis of factors associated with oral anticoagulant agent use among patients with nonvalvular atrial fibrillation.

	OACs not used		OACs used		*p*
	*N*	%	*N*	%	
Sex					0.002
Male	202	23.8%	646	76.2%	
Female	166	17.9%	763	82.1%	
Age					<0.001
<50 years	83	50.9%	80	49.1%	
50-75 years	193	17.6%	905	82.4%	
>75 years	92	17.8%	424	82.2%	
Hypertension					<0.001
No	142	33.6%	281	66.4%	
Yes	226	16.7%	1128	83.3%	
Diabetes mellitus					0.002
No	229	23.3%	752	76.7%	
Yes	139	17.5%	657	82.5%	
Current cigarette smoking					<0.001
No	282	18.4%	1250	81.6%	
Yes	86	35.2%	158	64.8%	
Type of atrial fibrillation					<0.001
Paroxysmal	229	34.0%	444	66.0%	
Nonparoxysmal	139	12.6%	965	87.4%	
Patient enrollment setting					<0.001
Outpatient	227	17.9%	1044	82.1%	
Inpatient	141	27.9%	365	72.1%	
Chronic kidney disease					0.440
No	330	20.5%	1282	79.5%	
Yes	38	23.0%	127	77.0%	
Stroke or systemic embolization					0.009
No	325	21.8%	1165	78.2%	
Yes	43	15.0%	244	85.0%	
Left ventricular ejection fraction					0.004
<40%	29	13.4%	187	86.6%	
≥40%	319	21.9%	1137	78.1%	
Heart failure					0.001
No	265	22.8%	895	77.2%	
Yes	60	14.3%	360	85.7%	
Ischemic heart disease					
No	265	22.8%	895	77.2%	
Yes	44	21.5%	161	78.5%	
Body mass index					0.288
<25	79	21.6%	287	78.4%	
≥25	240	19.1%	1018	80.9%	
CHA_2_DS_2_-VASc score					<0.001
<2	126	49.4%	129	50.6%	
≥2	242	15.9%	1280	84.1%	
CHA_2_DS_2_-VASc					<0.001
<3	180	36.8%	309	63.2%	
≥3	188	14.6%	1100	85.4%	

**Table 3:** Multivariate analysis of factors associated with OAC use among patients with nonvalvular atrial fibrillation.

Variable	CI	95% confidence interval		*p* value
Age (year)				
<50	1.0			
50-75	1.8	1.1	2.8	0.01
>75	1.4	0.8	2.3	0.24
Setting of patient enrollment (outpatient vs. inpatient)	1.9	1.5	2.6	<0.001
Current smoking (no vs. yes)	1.7	1.2	2.4	0.004
Cancer (no vs. yes)	1.8	1.0	3.0	0.04
Stroke or systemic embolization (yes vs. no)	1.7	1.1	2.5	0.01
Atrial fibrillation type (nonparoxysmal vs. paroxysmal)	3.2	2.5	4.2	<0.001
HAS-BLED score (per score increase)	0.8	0.7	0.9	<0.001
CHA_2_DS_2_-VASc score (≥2 vs. <2)	3.7	2.5	5.4	<0.001

## Data Availability

Readers can access the data supporting the conclusions of the study by contacting the corresponding author directly.

## References

[1] Lippi G., Sanchis-Gomar F. (2020). Global epidemiology of atrial fibrillation: an increasing epidemic and public health challenge. *International Journal of Stroke*.

[2] Ribeiro A. L., Otto C. M. (2018). Heartbeat: the worldwide burden of atrial fibrillation. *Heart*.

[3] López-López J. A., Sterne J. A. C., Thom H. H. Z. (2017). Oral anticoagulants for prevention of stroke in atrial fibrillation: systematic review, network meta-analysis, and cost effectiveness analysis. *BMJ*.

[4] Thomas K. L., Jackson L. R., Shrader P. (2017). Prevalence, characteristics, and outcomes of valvular heart disease in patients with atrial fibrillation: insights from the ORBIT-AF (Outcomes Registry for Better Informed Treatment for Atrial Fibrillation). *Journal of the American Heart Association*.

[5] Pan K. L., Singer D. E., Ovbiagele B., Wu Y. L., Ahmed M. A., Lee M. (2017). Effects of non-vitamin K antagonist oral anticoagulants versus warfarin in patients with atrial fibrillation and valvular heart disease: a systematic review and meta-analysis. *Journal of the American Heart Association*.

[6] January C. T., Wann L. S., Calkins H. (2019). 2019 AHA/ACC/HRS Focused Update of the 2014 AHA/ACC/HRS guideline for the management of patients with atrial fibrillation: a report of the American College of Cardiology/American Heart Association Task Force on Clinical Practice Guidelines and the Heart Rhythm Society. *Journal of the American College of Cardiology*.

[7] Borre E. D., Goode A., Raitz G. (2018). Predicting thromboembolic and bleeding event risk in patients with non-valvular atrial fibrillation: a systematic review. *Thrombosis and Haemostasis*.

[8] Boriani G., Proietti M., Laroche C. (2018). Contemporary stroke prevention strategies in 11 096 European patients with atrial fibrillation: a report from the EURObservational Research Programme on Atrial Fibrillation (EORP-AF) Long-Term General Registry. *Europace*.

[9] Magnani J. W., Norby F. L., Agarwal S. K. (2016). Racial differences in atrial fibrillation-related cardiovascular disease and mortality: the Atherosclerosis Risk in Communities (ARIC) Study. *JAMA Cardiology*.

[10] Chugh S. S., Havmoeller R., Narayanan K. (2014). Worldwide epidemiology of atrial fibrillation:a Global Burden of Disease 2010 Study. *Circulation*.

[11] Camm A. J., Fox K. A. A. (2018). Strengths and weaknesses of ‘real-world’ studies involving non-vitamin K antagonist oral anticoagulants. *Open Heart*.

[12] Bassand J. P., Virdone S., Goldhaber S. Z. (2019). Early risks of death, stroke/systemic embolism, and major bleeding in patients with newly diagnosed atrial fibrillation. *Circulation*.

[13] Azar R. R., Ragy H., Kozan O. (2020). Antithrombotic treatment pattern in newly diagnosed atrial fibrillation patients and 2-year follow-up results for dabigatran-treated patients in the Africa/Middle Eastern region: phase II results from the GLORIA-AF Registry Program. *Journal of the American College of Cardiology*.

[14] Morillo C. A., Banerjee A., Perel P., Wood D., Jouven X. (2017). Atrial fibrillation: the current epidemic. *Journal of Geriatric Cardiology*.

[15] Healey J. S., Oldgren J., Ezekowitz M. (2016). Occurrence of death and stroke in patients in 47 countries 1 year after presenting with atrial fibrillation: a cohort study. *Lancet*.

[16] al-Shamkhani W., Ayetey H., Lip G. Y. H. (2018). Atrial fibrillation in the Middle East: unmapped, underdiagnosed, undertreated. *Expert Review of Cardiovascular Therapy*.

[17] Chugh S. S., Roth G. A., Gillum R. F., Mensah G. A. (2020). Global burden of atrial fibrillation in developed and developing nations. *Global Heart*.

[18] Allan V., Honarbakhsh S., Casas J. P. (2017). Are cardiovascular risk factors also associated with the incidence of atrial fibrillation? A systematic review and field synopsis of 23 factors in 32 population- based cohorts of 20 million participants. *Thrombosis and Haemostasis*.

[19] Salam A. M. (2019). Atrial fibrillation in Middle Eastern Arabs and South Asians: summary of published articles in the Arabian Gulf. *Heart Views*.

[20] Apostolakis S., Zubaid M., Rashed W. A. (2013). Assessment of stroke risk in Middle Eastern patients with atrial fibrillation: the Gulf SAFE registry. *International Journal of Cardiology*.

[21] Salam A. M., AlBinali H. A., al-Mulla A. W., Singh R., Suwaidi J. A. (2013). Secular trends, treatments, and outcomes of Middle Eastern Arab and South Asian patients hospitalized with atrial fibrillation. *Angiology*.

[22] AlAwwa I., al-Hindi R., Alfraihat N. (2020). Prevalence and associated factors of undiagnosed atrial fibrillation among end-stage renal disease patients on maintenance haemodialysis: a cross-sectional study. *BMC Cardiovascular Disorders*.

[23] el Kadri M., Bazargani N., Farghaly M. (2019). Profiling clinical characteristics and treatment patterns among non-valvular atrial fibrillation patients: a real-world analysis in Dubai, United Arab Emirates. *Open Medicine Journal*.

[24] Hajj M., Ajrouche R., Zein S., Rachidi S., Awada S., al-Hajje A. (2020). Evaluation of risk factors and drug adherence in the occurrence of stroke in patients with atrial fibrillation. *Pharmacy Practice*.

[25] el-Deeb M. H., Sulaiman K. J., al Riyami A. A. (2014). 2014 Oman Heart Association protocol for the management of acute atrial fibrillation. *Critical Pathways in Cardiology: A Journal of Evidence-Based Medicine*.

[26] Zubaid M., Saad H., Ridha M. (2013). Quality of anticoagulation with warfarin across Kuwait. *Hellenic Journal of Cardiology*.

[27] Lip G. Y. H., Nieuwlaat R., Pisters R., Lane D. A., Crijns H. J. G. M. (2010). Refining clinical risk stratification for predicting stroke and thromboembolism in atrial fibrillation using a novel risk factor-based approach: the Euro Heart Survey on atrial fibrillation. *Chest*.

[28] Pisters R., Lane D. A., Nieuwlaat R., de Vos C. B., Crijns H. J. G. M., Lip G. Y. H. (2010). A novel user-friendly score (HAS-BLED) to assess 1-year risk of major bleeding in patients with atrial fibrillation: the Euro Heart Survey. *Chest*.

[29] Lip G. Y. H., Brechin C. M., Lane D. A. (2012). The global burden of atrial fibrillation and stroke: a systematic review of the epidemiology of atrial fibrillation in regions outside North America and Europe. *Chest*.

[30] Zubaid M., Rasheed W. A., Alsheikh-Ali A. A. (2011). Gulf Survey of Atrial Fibrillation Events (Gulf SAFE): design and baseline characteristics of patients with atrial fibrillation in the Arab Middle East. *Circulation. Cardiovascular Quality and Outcomes*.

[31] Li Y. G., Miyazawa K., Wolff A. (2019). One-year risks of stroke and mortality in patients with atrial fibrillation from different clinical settings: the Gulf SAFE registry and Darlington AF registry. *International Journal of Cardiology*.

[32] Borschel C. S., Schnabel R. B. (2019). The imminent epidemic of atrial fibrillation and its concomitant diseases - myocardial infarction and heart failure - a cause for concern. *International Journal of Cardiology*.

[33] Camm A. J., Amarenco P., Haas S. (2016). XANTUS: a real-world, prospective, observational study of patients treated with rivaroxaban for stroke prevention in atrial fibrillation. *European Heart Journal*.

[34] Alsheikh-Ali A. A., Omar M. I., Raal F. J. (2014). Cardiovascular risk factor burden in Africa and the Middle East: the Africa Middle East Cardiovascular Epidemiological (ACE) study. *PLoS One*.

[35] Boriani G., Proietti M., Laroche C. (2019). Association between antithrombotic treatment and outcomes at 1-year follow-up in patients with atrial fibrillation: the EORP-AF General Long-Term Registry. *EP Europace*.

[36] Xue Z., Zhang H. (2019). Non-vitamin k antagonist oral anticoagulants versus warfarin in Asians with atrial fibrillation: meta-analysis of randomized trials and real-world studies. *Stroke*.

[37] Bose A., O'Neal W. T., Wu C. (2019). Sex differences in risk factors for incident atrial fibrillation (from the Reasons for Geographic and Racial Differences in Stroke [REGARDS] Study). *The American Journal of Cardiology*.

[38] Nabauer M., Gerth A., Limbourg T. (2009). The Registry of the German Competence NETwork on Atrial Fibrillation: patient characteristics and initial management. *EP Europace*.

[39] Friberg J., Scharling H., Gadsboll N., Truelsen T., Jensen G. B. (2004). Comparison of the impact of atrial fibrillation on the risk of stroke and cardiovascular death in women versus men (The Copenhagen City Heart Study). *The American Journal of Cardiology*.

[40] Eikelboom J. W., O'Donnell M., Yusuf S. (2010). Rationale and design of AVERROES: apixaban versus acetylsalicylic acid to prevent stroke in atrial fibrillation patients who have failed or are unsuitable for vitamin K antagonist treatment. *American Heart Journal*.

[41] Letsas K., Karamichalakis N., Vlachos K. (2015). Managing atrial fibrillation in the very elderly patient: challenges and solutions. *Vascular Health and Risk Management*.

[42] Hindricks G., Potpara T., Dagres N. (2021). 2020 ESC Guidelines for the diagnosis and management of atrial fibrillation developed in collaboration with the European Association for Cardio-Thoracic Surgery (EACTS). *European Heart Journal*.

[43] Hu Z. C., Liu S. Y., Wu L. M. (2020). Factors influencing adherence to non-vitamin K antagonist oral anticoagulants in the early period after atrial fibrillation catheter ablation in China. *Chinese Medical Journal*.

[44] Xiang X., Cao Y., Sun K. (2018). Real world adherence to oral anticoagulant in non-valvular atrial fibrillation patients in China. *Current Medical Research and Opinion*.

[45] Liu T., Yang H. I., Gu L. (2020). Current status and factors influencing Oral anticoagulant therapy among patients with non-valvular atrial fibrillation in Jiangsu province, China: a multi-center, cross-sectional study. *BMC Cardiovascular Disorders*.

[46] Al-Shamiri M. (2020). Knowledge gaps about oral anticoagulant in Saudi patients. *International Journal of Pharmaceutical Research & Allied Sciences*.

